# Self-assembled microtubular electrodes for on-chip low-voltage electrophoretic manipulation of charged particles and macromolecules

**DOI:** 10.1038/s41378-022-00354-6

**Published:** 2022-02-28

**Authors:** Apratim Khandelwal, Nagendra Athreya, Michael Q. Tu, Lukas L. Janavicius, Zhendong Yang, Olgica Milenkovic, Jean-Pierre Leburton, Charles M. Schroeder, Xiuling Li

**Affiliations:** 1 Department of Electrical and Computer Engineering, University of Illinois Urbana-Champaign, Urbana, IL 61801 USA; 2 Nick Holonyak Micro and Nanotechnology Laboratory, University of Illinois Urbana-Champaign, Urbana, IL 61801 USA; 3 Department of Chemical Engineering, University of Illinois Urbana-Champaign, Urbana, IL 61801 USA; 4 Beckman Institute for Advanced Science and Technology, University of Illinois Urbana-Champaign, Urbana, IL 61801 USA; 5 Department of Electrical and Computer Engineering, Microelectronics Research Center, University of Texas, Austin, TX 78758 USA; 6 Coordinated Science Laboratory, University of Illinois Urbana-Champaign, Urbana, IL 61801 USA

**Keywords:** Microfluidics, Electrical and electronic engineering, Materials science

## Abstract

On-chip manipulation of charged particles using electrophoresis or electroosmosis is widely used for many applications, including optofluidic sensing, bioanalysis and macromolecular data storage. We hereby demonstrate a technique for the capture, localization, and release of charged particles and DNA molecules in an aqueous solution using tubular structures enabled by a strain-induced self-rolled-up nanomembrane (S-RuM) platform. Cuffed-in 3D electrodes that are embedded in cylindrical S-RuM structures and biased by a constant DC voltage are used to provide a uniform electrical field inside the microtubular devices. Efficient charged-particle manipulation is achieved at a bias voltage of <2–4 V, which is ~3 orders of magnitude lower than the required potential in traditional DC electrophoretic devices. Furthermore, Poisson–Boltzmann multiphysics simulation validates the feasibility and advantage of our microtubular charge manipulation devices over planar and other 3D variations of microfluidic devices. This work lays the foundation for on-chip DNA manipulation for data storage applications.

## Introduction

With the rapid proliferation of data, there has been a tremendous amount of research^1^ toward improving state-of-the-art data storage devices such as HDD-, SSD- and RAM-based technologies in terms of latency, power consumption, and storage density. Macromolecular data storage, such as DNA-based digital data storage, has thus emerged as one of the most promising means of achieving new forms of ultradense storage devices^2^. However, to contrast the current data storage mediums, DNA-based storage needs a precisely controlled and automated on-chip platform that exhibits controlled random access and rewriting and has high durability and appropriate error control^3^. Over the past years, advancements in micro and nanofabrication have enabled sophisticated on-chip devices and architectures capable of studying unique phenomena, including DNA-based storage more attainable. Integrated microfluidic devices with electronic^4,5^, optoelectronic^6,7^, magnetic^8,9^, thermal^10,11^, or acoustic^12^ functionality are well suited for serving as Lab-on-Chip (LOC) systems that enable a multitude of applications ranging from the characterization of minuscule liquid samples to running massive parallel bioassays. For the same reason, the field of electrokinetics has seen tremendous growth, as next-generation devices are able to provide more degrees of freedom toward assembly and manipulation of micro/nanosized particles; live cells^13,14^; and biomacromolecules, such as proteins^15^ and even DNA^16–18^. Three major electrokinetic phenomena^19^, namely, electrophoresis^20^, di-electrophoresis (DEP)^21^, and electroosmosis^22^, are the basis of micro/nanoparticle manipulation in aqueous solutions. DEP is applicable with both charged and neutral particles and can be useful to distinguish their behavior in either uniform or nonuniform electric fields. Electrophoresis uses an external electric field and measures the motion of charged particles relative to the liquid medium in which they are suspended. On the other hand, electroosmosis is the motion of a liquid that contains a net charge. In this study, we use the electrophoretic flux generated inside a microtube to manipulate charged particles.

There are several advantages of using electric fields for the on-chip manipulation of charged particles. Many parameters, including the magnitude and frequency of the signal, electrode distance, and electrode geometry, can be tuned to precisely control the field force exerted on the particles, which can help dictate the position of particles relative to electrodes or other particles in the system. Another major advantage of using an electric field stems from the ease of fabricating planar or 3D microelectrodes on chip. In this study, we demonstrate the use of 3D circular electrodes energized by DC voltage to manipulate and assemble particles on chip instead of relying on spontaneous fluid flow (microfiltration^23^ or inertial microfluidics^24^), optical traps^25^ or chemical modifications^26^.

Strain-induced self-rolled-up membranes (S-RuM), which were first discovered by Prinz et al. in 2001^27^, have proven to be an excellent option for various on-chip and off-chip applications in electronics, optics, materials science, biology, and micro/nanofluidics^28–37^. S-RuM tubes are formed by the spontaneous deformation of stressed thin films driven by the relaxation of strain energy. Membranes made of material combinations such as semiconductors^27,38^, oxides^39^, nitrides^40,41^, metals^42^, polymers^43^, and other hybrid thin films^44^ can be rolled up into micro- or nanotubes. The membranes can be strain engineered to precisely control tube diameter, tube length, number of turns and even tube orientation with high fabrication yields. In comparison with other 3D and 2D microscale designs on chip^45^, the tubular structure provides several inherent advantages. First, it provides a compact microfluidic chamber that can be modified for specific applications. The chamber can be made transparent for better optical imaging, integrated with electrodes for sensing and electrokinetics or mechanically transformed to yield structures suitable for self-assembly and storage^5,46^. Second, the tubular/cylindrical nature of the cuffed-in electrodes can provide a more uniform electric field, resulting in improved sensitivity and throughput compared to some of the planar and 3D variations of microfluidic devices. Third, the planar processing techniques used for fabricating these on-chip tubular devices are scalable to the industrial level and can virtually be fabricated on any substrate. Wafer-scale integration of these microtubular devices with multiple channels on chip can realize successful commercialization for more sophisticated needs in the field of flexible bioelectronics^47,48^. Moreover, once rolled, the footprint of the tubes can also be used for placing other microfluidic chambers on chip for integration purposes.

In this work, we focus on integrating the S-RuM platform, essentially a 3D microelectromechanical system (MEMS) approach, with an externally operated electrical circuit to control and manipulate charged particles and DNA molecules. It is of utmost importance to fabricate channel lengths compatible with the size of the species that needs to be manipulated so that the inherent charge on the species can be effectively accelerated (repelled/attracted) inside the channel and optimization is needed in terms of driving potential and channel dimensions to guide them. We believe our work will help pave the way for achieving this optimization by implementing a set of electrophoretic/electroosmotic control structures fabricated on chip using S-RuM technology and standard MEMS processing.

## Results and discussion

### Strain-engineered self-rolled-up membrane device

**Fig. 1:**
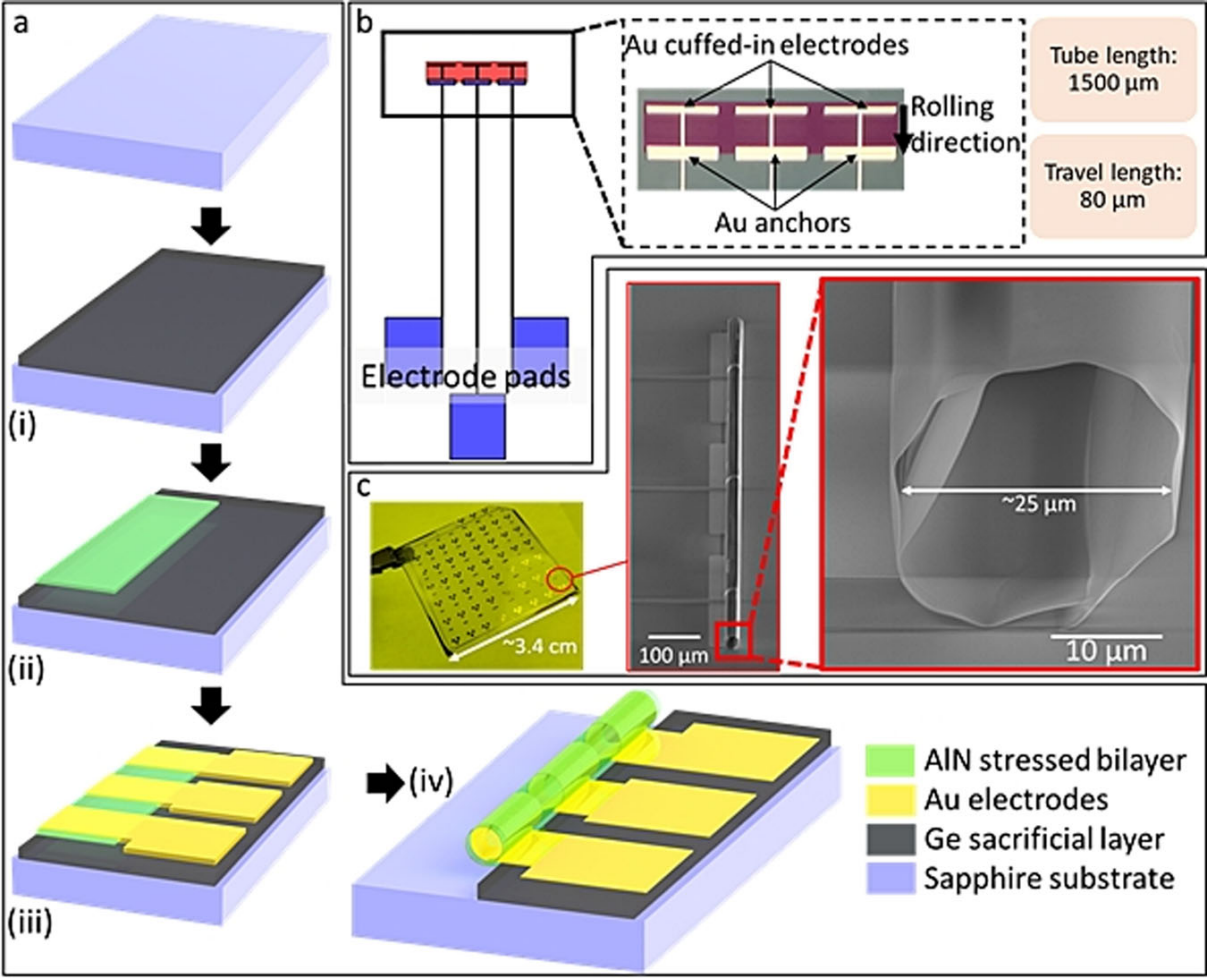
Design and fabrication of the 3D S-RuM microtube device. **a** Top to bottom, fabrication steps of the S-RuM microtube device: (i) e-beam evaporation of a sacrificial Ge layer on a transparent sapphire substrate, (ii) AlN stressed bilayer is then deposited by sputtering and patterned using photolithography, (iii) e-beam evaporation of the Au electrodes and patterning to the desired 2D geometry, (iv) selective gas-phase etching of Ge sacrificial layer to trigger the spontaneous rolling of the 3D S-RuM microtube device. **b** Schematic of the ‘unrolled’ device showing electrode pads and the optical image on the right showing the 2D geometry of Au electrodes to be cuffed-in once they are rolled-up. The total length of the tube is 1500 µm and the longitudinal travel length (gap between electrodes) for the particles is 80 µm. **c** Optical image of an array of microtube devices on chip. On the right side, a full and the zoomed-in SEM images of a microtube at a tilted angle showing the diameter (~25 µm) of the tube (note that the tube is not perfectly cylindrical because the metal electrodes in this study are not rolled along the entire tube wall in order to leave a window for imaging purposes)

An array of microtubes (S-RuM) were fabricated on chip via two-dimensional planar processing lithography. Metallic (Au) electrodes were deposited on the strain-engineered AlN membranes, which were then released from the substrate to roll-up and create a cuffed-in tubular electrode architecture. To simplify optical characterization, a semitransparent mixed phase (C to M) of sapphire substrates was chosen; however, there is no such technical limitation for using alternative insulating substrates such as glass or SOI substrates. The process steps for S-RuM fabrication are depicted in Fig. 1a and are also described in detail in the “Materials and methods” section. The Au electrodes are patterned so that they not only provide optimum voltage control and uniformity but also do not interfere with optical characterization (Fig. 1b). The axial configuration of electrodes enables a better definition and control of the electric field inside the microtubular channel compared to some of the other 3D (pillar- or sidewall-patterned electrodes) and planar versions of electrodes. It is worth noting that the current 2D geometry (I-shaped) of the electrodes is merely chosen to minimize the hindrance from the electrodes during optical measurements/imaging of inflow micro/nanoparticles. Much more control and uniformity can be achieved by rolling more complex 2D geometries, such as interdigital electrodes (Supplementary Note 1). Upon entry, microparticles experience considerable turbulence due to capillary forces that can significantly interfere with e-field control studies. Thus, the spacing between the electrodes (80 µm) and tube length (1500 µm) were optimized to minimize such effects and ensure that a fully developed flow was studied. The spacing between the electrodes is labeled as the travel length since it is the longitudinal distance the charged species inside the microtube needs to travel across under the effect of an e-field. The ‘internal’ nature of the electrode calls for DEP manipulation, but the three-electrode structure (left, middle, and right) merely serves as a proof-of-concept device for charged species manipulation, and there is no practical limitation on integrating more electrode feed lines inside the microtube or theoretical limitations for using more sophisticated manipulation techniques than electrophoresis.

The AlN-stressed bilayers were strain engineered to result in a specific 3D geometry of the microtube (see Supplementary Note 2 for details of the stress optimization process). The bilayer thickness (60 nm), cumulative stress (+400 MPa tensile membrane on top of a −1200 MPa compressive membrane) and patterned 2D geometry of the membrane are carefully controlled to yield tubes with a diameter of ~25 µm with ~1.5 turns (Fig. 1c). To further improve the yield and e-field interference of the microtubular devices, we selectively isolate the area of electrodes that lies outside the microtube using a thin (10 nm) alumina passivating layer. Thus, only the electrode area inside the microtube is in direct contact with the fluid of interest.

The overall 3D geometry of the S-RuM device, material properties of the constituents and axial nature of the electrodes all affect the sensitivity with which charged species can be manipulated to move inside the channel. In the subsequent section, the effect of the e-field on the particle position and velocity is demonstrated in detail using a polystyrene microsphere solution.

For the purpose of deterministically pumping and controlling the solution feed, it is imperative that the S-RuM platforms be encapsulated with a micro/nanofluidic circuit. The integration has three principal requirements: fabrication of micro/nanofluidic channels on a soft polymeric material such as polydimethylsiloxane (PDMS), aligning channels to the on-chip circuit and bonding the PDMS to the substrate to air seal the channels (prevent leakage). We have used conventional photolithography to strategically position the microtubes on the chip, thereby enabling encapsulation in PDMS-based microfluidics (Supplementary Note 3) to serve as an integrated device. Details on the microfluidic integration are described in the “Materials and methods” section.

### Particle dynamics and device utility

**Fig. 2:**
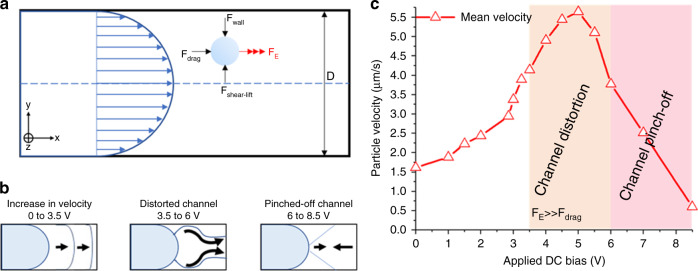
Particle dynamics inside the microtube device. **a** Schematic illustration showing a section of the microtube (diameter, D) with parabolic fluid flow profile, and a microparticle experiencing F_wall_ (repulsion force from the tube wall), F_shear-lift_ (shear gradient lift force), F_drag_ (viscous drag force) and an external F_E_ (electrostatic force) responsible for particle position and velocity inside the microtube. **b** Schematic illustrations (left to right) showing the transient behavior of particles between the electrodes inside the microtube. From 0 to 3.5 V, the velocity increases until the velocity profile becomes more uniform. From 3.5 V onward, the channel starts to distort and then is finally pinched-off when particle–particle repulsion interferes with F_E_. **c** Plot showing the increase in mean velocity of the particles with an applied bias (0 to 3.5 V). Beyond 3.5 V, the flow channel inside the microtube starts to distort and particles experience turbulence. The pinch-off condition is met when particles can no longer cover the distance between electrodes due to particle–particle repulsion resulting from the accumulated charged particles at the target electrode

Inside the microtubular channel, in addition to the viscous drag force (F_drag_) in the axial direction, the particles also experience two opposing transverse forces (the wall repulsion force (F_wall_) and shear-gradient lift force (F_shear-lift_)). Along the longitudinal direction of the microtube, the particles thus experience both axial and lateral forces compelling them to migrate toward equilibrium locations (Fig. 2a). For charged or polarizable particles, an additional external electrostatic force (F_E_) can be used to manipulate their position and velocity inside the channel. If a direct current (DC) field is applied tangential to the surface of the channel, a free particle within the electric double layer (EDL) will respond to the field and move toward the counter electrode. However, the EDL thickness in most channels carrying buffer solutions is much smaller than the characteristic lengths of typical microfluidic channels^49^. Thus, an electric field-mediated microtubular device enables more uniform linear electrokinetic motion inside the tubular channel (across the cross-section) by converting the supplied electric energy into kinetic energy for the microparticles. DC electric fields can be used to drive both fluid electroosmosis and particle electrophoresis inside microchannels; thus, particle manipulation in our electrophoretic manipulation study does not require hydrodynamic pumping of the particulate solution. A micropump is used for controlling the feeding of the particulate (1 µm polystyrene spheres) solution inside the tube (details in the “Materials and methods” section). The average velocity is then used to quantify the movement of charged particles inside the three electrode microtubes with respect to the applied bias. One end (left) of the microtube is loaded with a small volume of particulate solution until capillary action fills the entire tube. A specific mixture is prepared to ensure that the solution does not prematurely dry out while also helping minimize electrochemical effects on the electrode surface (details on the solution are provided in the “Materials and methods” section; see also Supplementary Note 4 for minimizing the electrochemical effects). Far from the point of entry, the microparticles are then allowed to stabilize before biasing the middle electrode and right-most electrode. The polystyrene spheres used in our study have an inherent negative zeta potential and thus travel toward the positive terminal. Figure 2b and c depicts a proportional response with increasing mean velocity as the applied bias is increased (up to 3.5 V). As expected, the particles accelerate, and the mean velocity in the channel increases with an increasing e-field. From 3.5 V onward, the off-center particles experience excessive electrical lift force, and the channel starts to distort. For particles in and around the channel axis, the e-field is symmetric along both the axial and transverse directions. However, for the off-center particles, e-field line distributions in the transverse directions become asymmetric. This asymmetricity results in a wall-induced electrical lift force causing the particle to drift away from the walls^50,51^. Moving from the center to the channel wall, this wall-induced electrical lift (channel distortion) becomes more pronounced with increasing DC field. Beyond 6 V, the excessive accumulation of negatively charged microspheres on the counter electrode counters the electrostatic force; thus, the channel is slowly pinched off as the average velocity of the particles diminishes. Supplementary Videos 1–4 show a slice of the real-time behavior of the particles affected by a DC electric field.

**Fig. 3:**
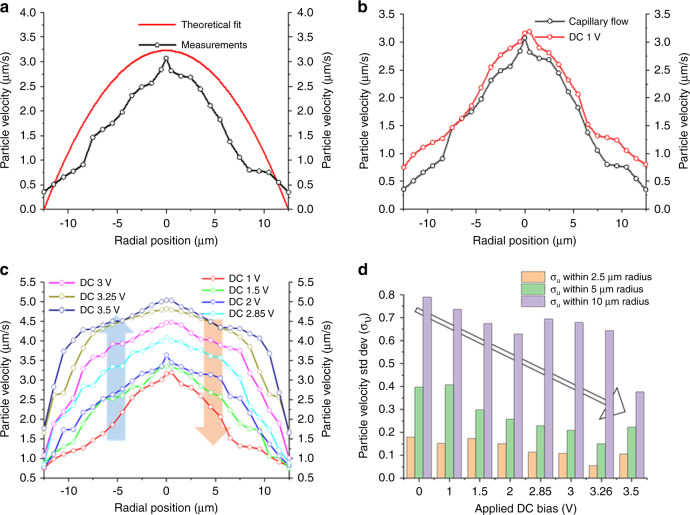
Manipulating particle velocity as a function of applied voltage. **a** Theoretical and experimental comparisons of the fully developed velocity profile of the microparticles inside the microtube (radial position) due to fluid flow. Note that the spheres on the tube walls (at ±12.5 µm) still have a nonzero velocity as they tend to roll on the inner-wall. **b** Change in the velocity profile (net increase in particle velocity) due to the external electrostatic force (F_E_). **c** Particle velocity as a function of applied bias. The blue arrow depicts the increasing magnitude of the applied bias and the orange arrow depicts the direction of the electric field inside the microtube. As the voltage is gradually increased, the velocity profile flattens **d** as evident from the downward trend (hollow black arrow) in the standard deviation of the particle velocity with respect to increasing bias. Moving further away from the center the difference is more noticeable due to edge effects

Inside a cylindrical channel, and sufficiently far from the point of entry, the radial velocity (*V*(*r*)) profile in a fully developed steady flow takes a parabolic form and can be described by the Hagen–Poiseuille equation:

$$V(r) = 2V_{\rm{m}}\left(1 - \left(\frac{r}{R}\right)^2\right)$$

where *V*_m_ is the mean velocity (calculated as 1.614 µm/s), *r* is the radial position from the center and *R* is the radius of the tube. The data in Fig. 3 are the result of tracking multiple particles over different frames, time intervals and under different bias conditions. For the purpose of making accurate measurements, it is critical to align the focal plane of interest to the imaging system. The effects of axial and angular misalignments are explained in detail in Supplementary Note 5. Figure 3a shows the comparison between the theoretical and experimental microparticle velocity profiles inside the 25 µm-diameter microtubular device under zero bias conditions. Applying a DC bias of 1 V increases the mean microparticle velocity; however, the velocity profile is still nonuniform with respect to the radial position, as shown in Fig. 3b. As the applied bias is gradually increased, the turbulency in the flow increases, and the particle velocity profiles become much more uniform over most of the flow (Fig. 3c). A much sharper change in velocity is observed near the boundary because of the no-slip condition on the channel walls. In a geometrically nonuniform microchannel, the breakdown in e-field symmetry can lead to di-electrophoretic particle motion effects when exposed to a DC electric field, and the influence of the insulating channel wall can be significantly difficult to curb. Most traditional microfluidic channels are rectangular, and these edge effects become more prominent as the particles move in transverse directions^52^. Therefore, the axial and tubular nature of the electrodes inside a circular cross-section channel can minimize such effects and lead to more uniform fluid fronts. As shown in Fig. 3d, in the vicinity of a radius of up to at least 5 µm, the standard deviation in particle velocity gradually decreases until an optimum e-field condition is reached. The effects of a greater magnitude of DC fields were explained in earlier discussions (Fig. 2c). Deviations from traditional electrokinetic phenomena can also be understood by examining the effects of the e-field on the electrophoretic mobility of the PSB microspheres (1 µm). Supplementary Note 6 explains in detail the effects of the e-field and particle velocity on the electrophoretic mobility. Additionally, we used 200 nm polystyrene latex spheres to demonstrate bulk manipulation, and the corresponding video still-frames and fluorescence microscopy images can be found in Supplementary Note 7.

**Fig. 4:**
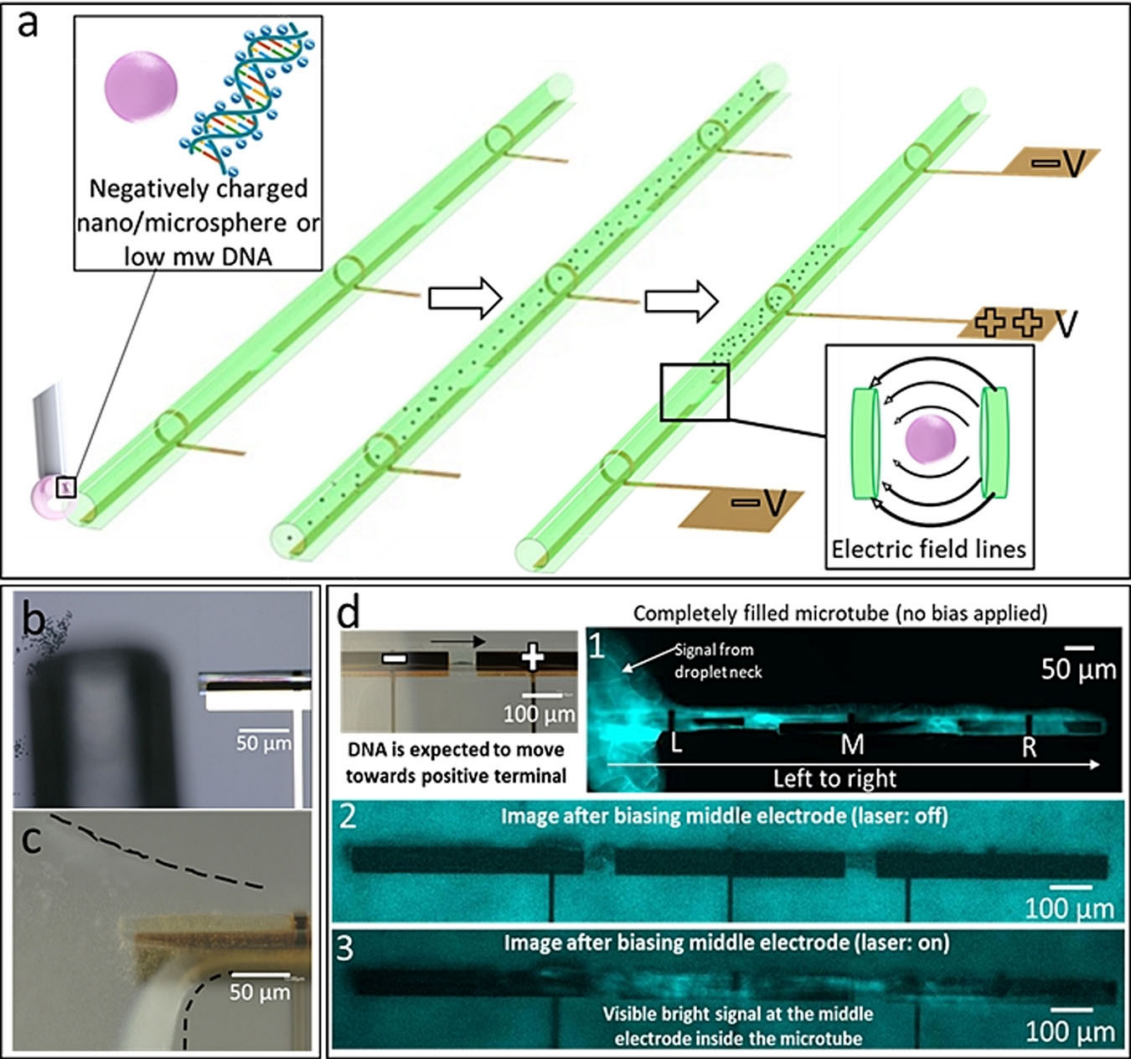
Device used for the DNA capture/hold/release mechanism. **a** 3D schematic showing the tubular device being filled with species containing charged particles (nano/microsphere and DNA in our case). On the right, when the capillary actions takes place and uniformly fills the tube with charged species, a low positive voltage is applied on the middle electrode and a low negative voltage is applied on the outer electrodes to capture and hold the charged species inside the tube. **b** Optical image showing the microsyringe needle strategically placed near the channel end and close to the tube to easily dispense fluids. **c** Optical image showing the capillary neck formed at the tube opening after dispensing the fluid. **d** A low potential difference of ~2 V is sufficient to move DNA from the left to middle electrode. The 1, 2, and 3 labels are fluorescence microscopy images serving as the proof for localizing DNA toward the middle electrode by appropriately biasing the electrodes

Large-scale macromolecular (DNA)-based data storage relies heavily on laborious manual pipetting of DNA solutions for synthesis and microanalysis. An automated lab-on-a-chip (LOC) platform would contribute significantly to industrial-level scalability for DNA-based digital data storage. The use of Electrophoresis to drive the sequential capture, holding and release of DNA molecules on chips requires the integration of electric field-mediated trapping devices^16^. Given the diameter of DNA (~2 nm), such devices would require compatible channel sizes, integrated electrodes, and uniform e-fields for the precise control of DNA movement inside the channel. In our study, we demonstrate the utility of manipulating charged species inside a closed tubular structure, and as shown in Fig. 4, we extend our utility by trapping low molecular weight (mw) DNA inside the microtube by appropriately biasing the cuffed-in electrodes, which has been recently proposed by Athreya et al.^53^. The strategic loading and DNA capture mechanisms are depicted in Fig. 4a. The low mw-labeled DNA used in our study has a net negative charge and can be located optically using laser excited fluorescent microscopy. A more positive voltage is thus applied on the middle electrode to drive, capture, and concentrate DNA in the center of the tube. The tubular electrode geometry provides uniform field lines between the parallel electrodes that facilitate holding DNA inside the channel. The optical and fluorescence microscopy images in Fig. 4b–d highlight the artifacts associated with DNA loading, manipulation, and detection, respectively. We believe that a 3D platform such as S-RuM built on 2D planar processing can be optimized to provide a much more uniform electric field and thus better manipulate DNA inside integrated electronic devices with nano- or microscale resolution compared to microfluidics based on nontubular electrode designs. In the subsequent section, to support our argument, we carried out dynamic simulations for microfluidic devices carrying DNA buffer solutions and compared e-field profiles inside a tubular design (such as S-RuM) with their planar counterparts.

### Validation using multiphysics simulations

**Fig. 5:**
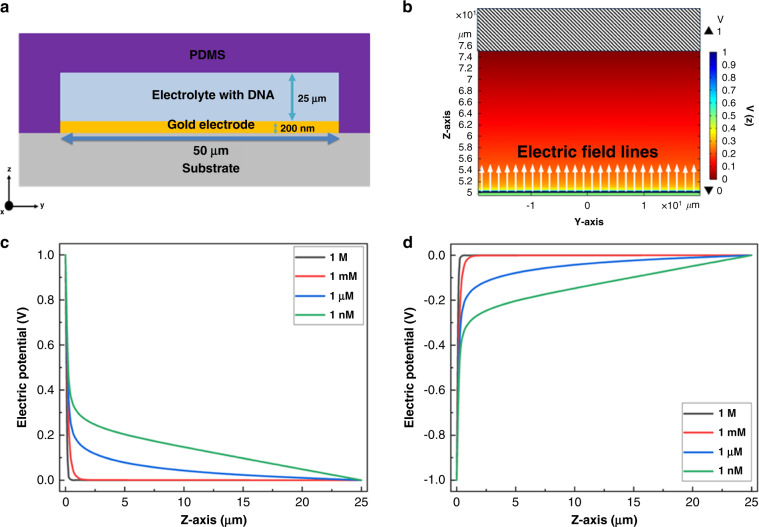
Electrostatic potential dynamics inside the designed planar electrode device. **a** Cross-section of the device in the z–y plane. **b** Electric potential with field lines inside the tunnel with 1 nM KCl solutions at 1 V bias. **c**, **d** Electric potential along the height of the tunnel with different voltage biases applied to the gold electrode: **c** −1 V and d 1 V

To effectively manipulate the flow of DNA under the influence of DC-biased electrodes, the electrolyte screening length (or Debye length), which is inversely proportional to the square root of the electrolyte concentration, is of central importance. Hence, the sizes of the channels carrying the DNA strands should be optimized along with the electrolyte concentration to avoid any leakage of DNA strands. We first simulated the device structure with planar electrodes depicted in Fig. 5a^53^. The structure consisted of a 50 µm × 100 µm alumina substrate onto which a 200 nm × 25 µm gold electrode was deposited. This setup was encapsulated with polydimethylsiloxane (PDMS), leaving a tunnel height of 25 µm. COMSOL Multiphysics simulation that coupled the continuity equation for the concentration of ion species in the electrolyte with Poisson’s equation (detailed description in the “Materials and methods” section) was used to obtain the electrostatic potential inside the tunnels (as shown in Fig. 5b). The electric field lines point upward inside the tunnel.

Figure 5c and d show the electrostatic potential profiles inside the tunnel with applied voltage biases of 1 V and −1 V, respectively, and for electrolyte concentrations inside the tunnels varying from 1 M to 1 nM. Here, one sees a steep drop in the potential across the electrode surface followed by an exponential decrease from the electrode-electrolyte interface to the far end of the tunnel. For 1 M KCl, the potential drops down to 0 V within a few nanometers from the interface. However, the electric potential at the far end (z = 25 µm) of the tunnel decreases to 0 V, which creates a path for the chimeric DNA strands to escape from the tunnel.

**Fig. 6:**
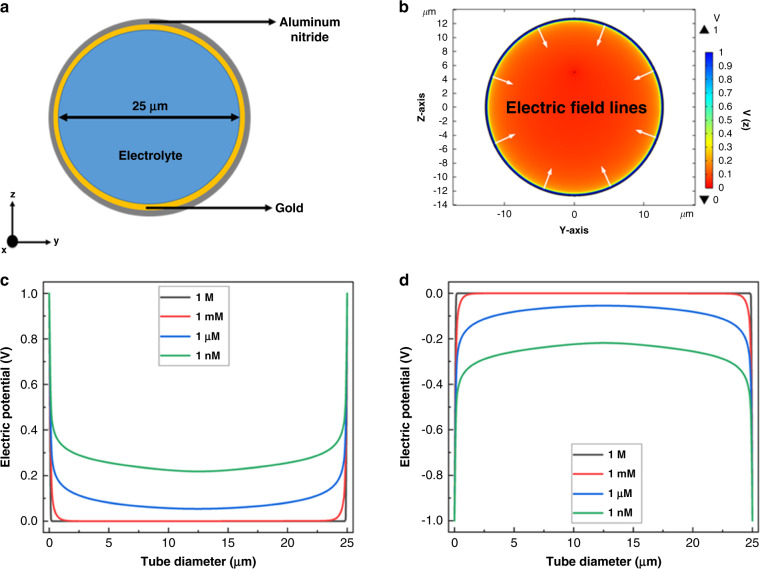
Electrostatic potential dynamics inside the designed tubular electrode device. **a** Cross-section of the rolled tube in z–y plane. **b** Electric potential with field lines inside the tube with 1 nM KCl solutions under 1 V bias. **c**, **d** Electric potential along the diameter of the rolled tube under different voltage biases applied at the gold electrode: c −1 V and d 1 V

An encouraging solution to the problem is to use a closed device design, such as a tubular structure where electrodes cover all walls of the tunnels, resulting in a uniform radial electric field inside the channel, as depicted in Fig. 6a. Figure 6a and b shows the device structure of rolled-up aluminum nitride tubes with cuffed gold electrodes to control the flow of DNA and its electrostatic potential inside the tunnels, respectively. One can observe that the electric field lines point inwards inside the tube, creating a uniform and concentrated potential.

Figure 6c and d show the electrostatic profile inside the tube for applied voltage biases of 1 V and −1 V, respectively, with different electrolytic concentrations inside the tube. The electric potentials with lower electrolyte concentrations, such as 1 µM and 1 nM, remain at ~ ±100 mV and ~ ±300 mV, respectively, at the center of the tube. This clearly indicates that the motion of biomolecules, such as DNA, can be effectively controlled by the voltage applied to the cuffed electrodes in the S-RuM device structures.

In summary, we report a versatile on-chip technique for controlling the particles in microchannels with a 3D electric field. The highly scalable and compatible planar processing techniques reported in our study can be used to incorporate different materials while also optimizing the tubular geometry to accommodate numerous applications in the field of micro/nanofluidics. The compact structure not only provides precise fluid control but also reduces sample consumption; additionally, the strong confinement effects enable efficient particle manipulation. Lower voltages (<2–4 V) can be used to generate a much higher e-field (>10^4^ V/m) between the electrodes. This lowers the power consumption and reduces the Joule heating effect that is dominant in other designs of 3D electrophoretic devices that have operating voltages as large as >10^3^ volts. Moreover, the current design can easily be adapted for DEP-based manipulation techniques that would require an even lower peak-to-peak working voltage. Successful encapsulation of S-RuM devices with traditional microfluidics will further encourage integration with commercially available automated DNA sequencing nanopore technology. Once integrated into LOC systems, such electrokinetic manipulations of charged species can result in easy operation and reconfiguration for application in numerous other technologies, such as point-of-care applications. Compared to traditional planar versions, our demonstrated 3D microtubular device design is shown to be a highly promising platform for efficient manipulation of charged species, including DNA molecules, for data storage.

## Materials and methods

### AlN microtube fabrication with cuffed-in electrodes

The AlN microtubes used in this study were fabricated with a previously established S-RuM fabrication technique. A 100 nm Ge layer was deposited on a mixed phase 4-inch sapphire wafer using e-beam evaporation (Kurt J. Lesker Metals E-Beam Evaporator). Then, the AlN-stressed bilayer was deposited using RF magnetron reactive sputtering. Die-sized samples were cleaved and diced (FlipScribe 100, LatticeGear) from the four-inch wafers. Contact photolithography (AZ 5214E dual tone photoresist and Karl Suss MJB3 Contact Mask Aligner) was then used to pattern arrays of rectangular mesas defined using reactive ion etching (Oxford Mixed ICP-RIE). Subsequent negative lithography was used to lift off (AZ-917 MIF) 185 nm of cumulative Ti/Au metal deposited using e-beam evaporation. The metal also acted as an anchor for the microtubes. A thin (5 nm) conformal cover layer of Al_2_O_3_ was deposited using atomic layer deposition (Veeco NanoTech Atomic Layer Deposition tool) on top to facilitate the rolling process and maximize the yield. The exposed electrode area (area outside the tube) was also coated with a thin layer (10 nm) of ALD alumina. Another lithography step was performed to open the electrical contacts and etch windows on one end of the rectangular mesa to define the rolling front (more information on etching the windows and ALD passivation can be found in Supplementary Note 8). The Ge underlayer was then selectively dry-etched (a Xactix XeF_2_ etching system) using fluorine-based chemistry to release the membrane from the substrate, which eventually rolled up into a microtube with cuffed-in electrodes. The tube windings, tube diameter, and electrode area were engineered in a specific way to facilitate both capillary action and postprocessing fluorescent imaging.

### Integration with traditional microfluidics

Standard soft lithography processes were used for the fabrication and encapsulation of microfluidic channels. A PDMS mixture of elastomeric polymer and crosslinking agent at a weight ratio of 10:1 was used to cast the channels. A master mold for PDMS molding was fabricated on a 2-inch Si wafer by patterning SU-8 negative photoresist (SU-8 25, MicroChem Corp., USA). The PDMS mixture was then poured on top of the mold followed by degassing (1.5 h) and curing (at 60 °C for 24 h). After curing, the PDMS was peeled off, and tubing connections were made by punching holes into channel ends located on PDMS. The sapphire substrate was then exposed to a vacuum ultraviolet (VUV) ozone environment (custom setup) to enhance bonding with PDMS. Finally, the PDMS slab was aligned using a Karl Suss MJB3 contact mask aligner. External elastic tubing connections were then made to test the device.

### Polystyrene solution preparation

The 1 µm polystyrene beads (PSB) (Alfa Aesar) were ultrasonicated for 15 min prior to use. To assist in wetting the tubes and prevent the solution from drying, 1 volumetric part of PS bead solution (10 wt% suspension in water) was mixed with 8 parts deionized water (DI), 3 parts isopropyl alcohol (IPA), and 3 parts propylene glycol (PG) [8:3:3:1, DI:IPA:PG:PSB]. Additionally, a solution was prepared without IPA and propylene glycol (8:1, DI:PSB) to compare and contrast the effects of evaporation and electrochemical reaction on thee electrodes. In both cases, the dispensing solution was thoroughly shaken for 2 min prior to use. A 100 µl NanoFil syringe pump (World Precision Instruments UMP3) was used to automatically dispense the solutions (volume of 500 nl).

### Charge particle manipulation and fluorescent imaging

For fluorescent beads, 200 nm polystyrene latex beads (Invitrogen) were used as received. For DNA imaging, a 10 ng/µL solution of a low molecular weight DNA ladder (25–766 bp, New England Biolabs) was stained at a 1:10 dye:base pair ratio using an intercalating nucleic acid dye (YOYO-1, Invitrogen) in 1X TAE buffer comprised of 40 mM Tris (pH 7.6), 20 mM acetic acid, and 1 mM EDTA. The concentration of DNA was checked via a UV–Vis spectrophotometer (Nanodrop) prior to dilution to the desired concentration. The solution containing DNA and YOYO-1 was incubated in the dark for at least 2 h prior to imaging.

The bead solution was diluted to 1/8th of the as-received concentration (2 wt%). The diluted nanobead solution/DNA-containing solution was drop-cast on one end of the microtube, and sufficient time was given to let capillary action take place and the liquid stabilize/settle inside the tube. Note that the outside of the tube also had a considerable amount of liquid sticking to the tube surface due to surface tension and static charge effects. Then, a constant DC voltage was applied on the right-most electrode pad in reference to the left-most electrode to induce charge particle/DNA movement. The potential was kept constant until the liquid dried off. The 2D mask designs for the cuffed-in electrodes were designed so that when the tube was flipped, the electrodes did not interfere with imaging.

Epi-illumination of the fluorescent beads and labeled DNA was achieved using an inverted microscope (Olympus IX71) equipped with a 488 nm, 50 mW continuous wave (CW) laser (Excelsior-488, Spectra Physics). The incident beam was passed through a 488 nm longpass beamsplitter (Chroma), and the emitted light was filtered through a 525/50 nm bandpass filter (Semrock), where it was captured by an electron-multiplying charge-coupled device (EMCCD, Andor) camera. A ×10 0.25 NA objective (Olympus) was used, resulting in an overall pixel size of 1.6 µm.

### Particle velocity measurements

An ultrazoom optical microscope (Keyence VHX 7000 series) was used with a partial ring illumination filter to capture 2048×1536 pixel (resolution) videos at 15 fps. The RGB coded videos were analyzed using open-source image analysis software (ImageJ). A TrackMate-ImageJ extension was used to track more than 30 particles across the traveling length (electrode gap) under different biasing conditions. The initial and final positional coordinates of the particles were extracted for different focal planes (radial position) across a depth of 25 µm (tube diameter). The extracted pixel coordinates of the particles were used to calculate the distance traveled across a span of 5 to 6 seconds (77 or 92 frames, respectively).

### Use of COMSOL multiphysics

We performed our simulations using COMSOL Multiphysics software, combining the transport of diluted species with electrostatic physics to account for mass transfer and charge transfer. One of the simplest physical models of a double layer is the Gouy-Chapman-Stern (GCS) model, where the diffuse double layer is treated as a multiphysics coupling of the Nernst–Planck equations for the mass transport of all ions, with the Poisson equation (Gauss’s law) being used for the charge density and electric field. The combination of these equations, referred to as the Poisson-Nernst Planck (PNP) equations or Nernst–Planck-Poisson (NPP) equations, was used to solve for the potentials induced by the electrodes inside the channels.

We use COMSOL finite element modeling software to simulate electrically controlled nanofluidic devices with two-dimensional (2D) geometry. The fluxes of electrolytic ions, $J_i$, in the nanofluidic channel were modeled using the Nernst-Plank equation, as described in Eq. 1:

1 $$J_i = - D_i\nabla c_i - u_{m,\;i}z_iF_{c_i}\nabla \phi \,\left[ {{\rm{SI}}\;{\rm{Unit}}:\frac{{mol}}{{m^2.s}}} \right]$$

where $D_i$ is the diffusion coefficient of the ion species $i$, $c_i$ is the concentration of the polar ions in the electrolyte ($i$ = + or − with $z_i$ = +1 or −1 charge, respectively), $u_{m,i}$ is the mobility, $F$ is the Faraday constant, and $\phi$ is the electric potential obtained from Poisson’s equation (Eq. 2):

2 $$\nabla .\left( { - \varepsilon \nabla \phi } \right) = \rho\, [SI\;Unit:V]$$

where $\varepsilon$ is the relative permittivity and $\rho$ is the charge density, which depends on the ion concentration, $\rho = F\left( {c_ + - c_ - } \right)$.

The outer boundaries of the devices were set to ground conditions in which $\phi = 0$. The ion concentrations were set to their bulk values, which were electroneutral. Additionally, we assumed that there were no ion reactions in the electrolyte. Hence, the conservation of mass for both ion species required that $\nabla .J_i = 0$.

To implement the Stern theory of double-layer formation, we imposed a boundary condition for the electrolyte potential at the electrode interface having a constant Stern layer thickness, $\lambda _S$, as described in Equation 3:

3 $$\phi + \lambda _S\left( {n.\nabla \phi } \right) = \phi _M$$

where $n$ is the unit vector normal to the electrode surface and $\phi _M$ is the potential applied to the electrode.

## Supplementary information

Supplementary Document_Revised

Supplementary Document

Supplementrary video_1

Supplementrary video_2

Supplementrary video_3

Supplementrary video_4
