# Supplementary material for: Self-assembled microtubular electrodes for on-chip low-voltage electrophoretic manipulation of charged particles and macromolecules
Source: Microsyst Nanoeng. 2022 Feb 28;8:27. doi: 10.1038/s41378-022-00354-6 (PMC8882674; doi:10.1038/s41378-022-00354-6)
Supplement: Supplementary file 2 — Supplementary Document [file 41378_2022_354_MOESM2_ESM.docx]

Supplementary Information

**Self-Assembled Microtubular Electrodes for on-Chip Low-Voltage Electrophoretic Manipulation of Charged Particles and Macromolecules**

Apratim Khandelwal^1,2^, Nagendra Athreya^1,2^, Michael Q. Tu^3,4^, Lukas Janavicius^1,2^, Zhendong Yang^1,2^, Olgica Milenkovic^1,5^, Jean-Pierre Leburton^1,2^, Charles Schroeder^3,4^, Xiuling Li^1,2,4*^

^1^Department of Electrical and Computer Engineering, ^2^Nick Holonyak Micro and Nanotechnology Laboratory, ^3^Department of Chemical and Biomolecular Engineering, ^4^Beckman Institute for Advanced Science and Technology, ^5^Coordinated Science Laboratory, University of Illinois Urbana-Champaign, Urbana, Illinois 61801, United States

[*Corresponding author: xiuling@illinois.edu](mailto:*Corresponding%20author:%20xiuling@illinois.edu)

**Supplementary Note 1**

There are no technical limits on rolling other complex 2D electrode geometries (example Fig. S1).

**Supplementary Note 2**

The following three thin-film parameters were optimized to carefully manipulate the outer diameter of the microtube as per the needs of our experiments: (i) tensile/compressive stress in the AlN bilayer, (ii) thickness of the AlN bilayer, (iii) 2D geometry and thickness of the metal layer. Effects of the later are already explained in the previous supplementary note. However, thicker the metal, harder it is for the membrane to roll-up and thus beyond a certain thickness (>250 nm) the diameter of the microtube will be substantially changed. For our experiments, we kept the metal thickness around 180 nm and created a process by which we could control the outer diameter of the microtube by manipulating the stress in AlN bilayer. The table and figure below summarize the stress optimization study. A more detailed study is published in several of our other works^1^.

The diameter of the roll-up membrane can be directly related to its thickness and Young’s modulus.

D ∝ $\frac{t}{E}$

Where, D is the diameter (µm), t is the cumulative membrane thickness (nm) and E is the Young’s modulus (GPa) of the membrane (stress per unit strain in the membrane). We found out that, a 1500 x 250 µm^2^ AlN mesa with a bilayer stack of 30 nm tensile (+400 MPa) AlN membrane on a 30 nm compressive (-1200 MPa) AlN membrane covered with effective electrode area of approximately 63600 µm^2^ (~ 21200 µm^2^ per electrode) rolls-up into a microtube of diameter close to 25 µm. Once a diameter was chosen, the rolling length was optimized to result in a perfect 1.5 winding tube to prevent any leakage.

**Supplementary Note 3**

Details on microfluidic encapsulations are provided in methods section. Fig. S3a shows the schematic demonstrating the microfluidic encapsulation along with optical images of the mold (Fig. S3b), PDMS containing channel markers (Fig. S3c), final device (Fig. S3d) and microtube inside channel (Fig. S3e).

**Supplementary Note 4**

Viscosity, capillary, ability to suppress bubbling at high voltage, maybe schematic, still frames from hydrolysis (on planar electrodes). Refutas and Chevron formulation^2^ was used for the theoretical estimation of the viscosity of the liquid mixture (8 parts de-ionized water^3^ (DI), 3 parts isopropyl alcohol^4^ (IPA), and 3 parts Propylene glycol^5^ (PG)). First, the viscosity blending number (VBN) of each liquid component is calculated which is then used to determine the VBN of the liquid mixture as shown below.

$${VBN}_{x}=\frac{\ln\eta_{x}}{\ln\left( 1000*\eta_{x} \right)}$$

The VBN of each volumetric liquid component was then used to calculate the VBI of liquid mixture (results summarized in Table S1),

$$\text{VBN}\text{mixture}=\sum_{k=0}^{n} {(V}_{x})(VBN)$$

Glycol in the mixture was added to increase the viscosity and thus helped to reduce the volumetric flow rate according to Hagen-Poiseuille equation (volumetric flow rate is inversely proportional to viscosity of the liquid mixture).

Glycol also helps in reducing the vapor pressure of the liquid mixture. This slowed down evaporation and allowed for liquid to last longer under the illuminated light source for imaging. Generation of gas bubbles (H_2_) due to the electrolysis reaction at electrodes can seriously disrupt the electrophoretic migration^6^. Image below shows the electrolysis reaction induced bubbling on planar electrodes covered with a ionic salt solution (>90wt% water). Adding glycol to the mixture suppresses the bubble formation up to a certain limit of the applied voltage (<9V).

**Supplementary Note 5**

Perfect optical alignment with the plane of interest (inside the microtube) can be challenging. Our optical imaging set-up was mounted on a motorized X-Y-Z control stage. Control over vertical height (z plane) is important to make sure the frames are being grabbed from the central plane of the tube. Due to the small working distance, a slight change in the z-height can result in a deviation of Δr away from central plane. In addition, optical axis of the microscope may not be perfectly perpendicular to the plane of the tube. This would introduce a tilt angle between the two. Effects of axial and angular misalignments on the velocity profile can be understood from the equation below^7^.

$$V \left( r \right)=2V_{m}\left[ 1-\left( \frac{\Delta r}{R} \right)^{2} \right]\left[ 1-\frac{r^{2}}{R^{2}-{\Delta r}^{2}} \right]\cos\theta$$

Out of plane components were eliminated from the measurements by calculating a nominal radius (R_nom_) of the microtube. Off-center focal planes were recognized by calculating Δr using nominal tube radius (equation below).

$$\Delta r=\sqrt{R^{2}-R_{nom}^{2}}$$

Efforts were made to minimize Δr and θ. Since the tilt angle θ is very small, its cosine converges to unity. Also, for small changes in nominal radius, effects of Δr can be neglected thus giving us the simplified form of velocity profile equation:

$$V \left( r \right)=2V_{m}\left( 1-\left( \frac{r}{R} \right)^{2} \right)$$

**Supplementary Note 6**

DC electric field is generated between the circular electrodes separated by a distance of 80 µm. This DC electric field imparts kinetic energy to the solution and the particles which helps perpetuate both fluid electroosmosis and particle electrophoresis inside the microtube. In the microfluidics community, these two phenomena are often combined into one term called linear electrokinetic motion^8^. Assuming a particle in the velocity front inside the microtube as shown in Fig. S4, we can estimate its instantaneous position (r_p_) inside the microtube, with respect to time (t; t_1_ to t_2_) as,

$$r_{p}=\int_{t_{1}}^{t_{2}} v_{p}\left( t \right) dt$$

where, $v$ _p_ is the particle velocity. The wall-induced repulsive forces can be neglected on particles traveling along center line (z-axis) since the particle size (1µm) is significantly smaller than channel size (25µm). Assuming the particle-particle interactions are negligible, under the thin EDL (electric double layer) limit, the particle migration velocity (V_p_) can be given as^9^,

$$V_{p}=\mu_{ep}E$$

where, µ_ep_ is the electrophoretic mobility and E is the applied electric field.

Particles close to the wall will experience wall-induced repulsive force and lean towards non-linear electrokinetics (Fig. S5a). Since the electrophoretic velocity is proportional to the applied e-field, theoretically, the electrophoretic mobility is not expected to be affected by applied e-field. The electrophoretic mobility of particles traveling along the centerline is calculated and plotted in Fig. S5b. Mean velocity is cumulative function of both centerline and off-center particles and thus mean velocity derived electrophoretic mobility shows strong dependence on either particle velocity or applied e-field (Fig. S5c,d). As shown in Fig. S5b, true nature of electrophoretic mobility is seen in the flattened, uniform velocity profile regime (2.85 V to 3.5 V).

**Supplementary Note 7**

Our microtube device was also used to show micromanipulation of Latex nanospheres, 200nm in diameter. Bulk manipulation was studied since it was significantly harder to resolve individual particles using our imaging set-up. First, on the left and right end of the tube we drop casted droplets containing 200 nm negatively charged spheres. Enough time is spared to allow capillary action to take place and the liquid to stabilize and settle down inside the tube. Note that the outside of the tube also has considerable amount of liquid sticking to the tube surface due to surface tension and static charge effects. A constant 2V DC bias is sequentially applied on the middle, right and left electrode to move the nanospheres from one end to other inside the microtube. Video still frames showing nanosphere bulk accumulation on middle, right and left electrode positions with respect to applied bias are shown in Fig. S6. Fluorescent imaging is performed for further confirmation and to examine the areal distribution of the charged spheres inside the microtube (Fig. S7).

**Fig. S1: 2D image of interdigital electrodes and SEM of rolled-up tubes array.**


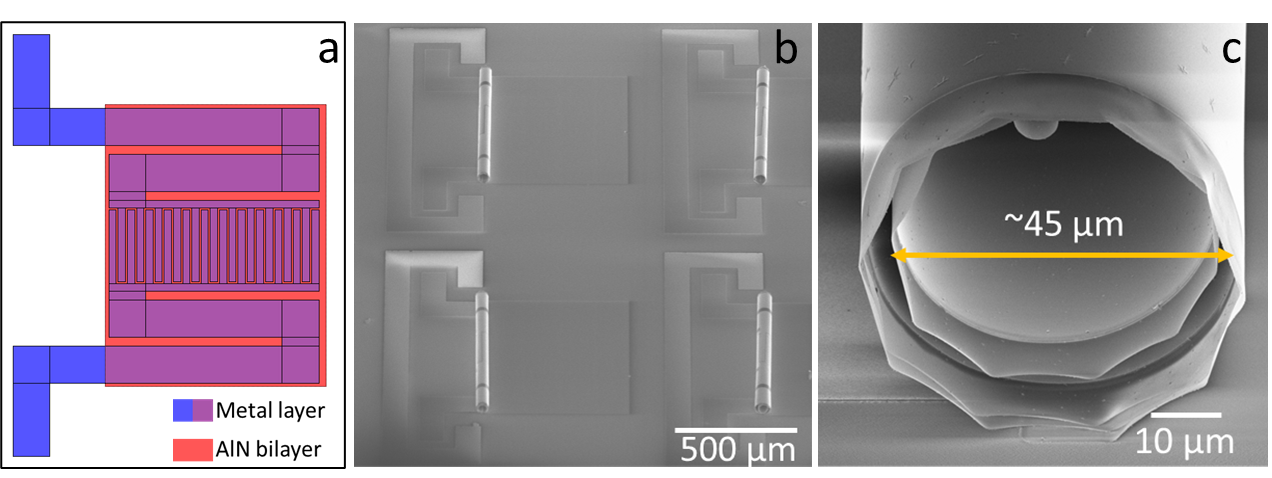


**a**, 2D mask pattern of the inter-digital electrode design. **b**, SEM image of the array of multi-turn (3.5 windings) microtubes with interdigital cuffed-in Au electrodes. These can be easily integrated with traditional microfluidics to create a linear array device. **c**, Zoomed-in SEM image of a microtube with cuffed-in interdigital electrode design. The electrode design for the DNA capture microtube (diameter, 25 µm) occupies much less real estate on the AlN bilayer compared to the interdigital electrode design and thus the diameter (or the channel size, 45 µm) of a rolled-up interdigital electrode design is substantially larger. An interdigital electrode design could provide more degrees of freedom and surface area, especially for applications pertaining to on-chip macromolecular sensing and storage.

**Fig. S2: Diameter of the as-rolled AlN stressed bilayers as a function of membrane stress.**


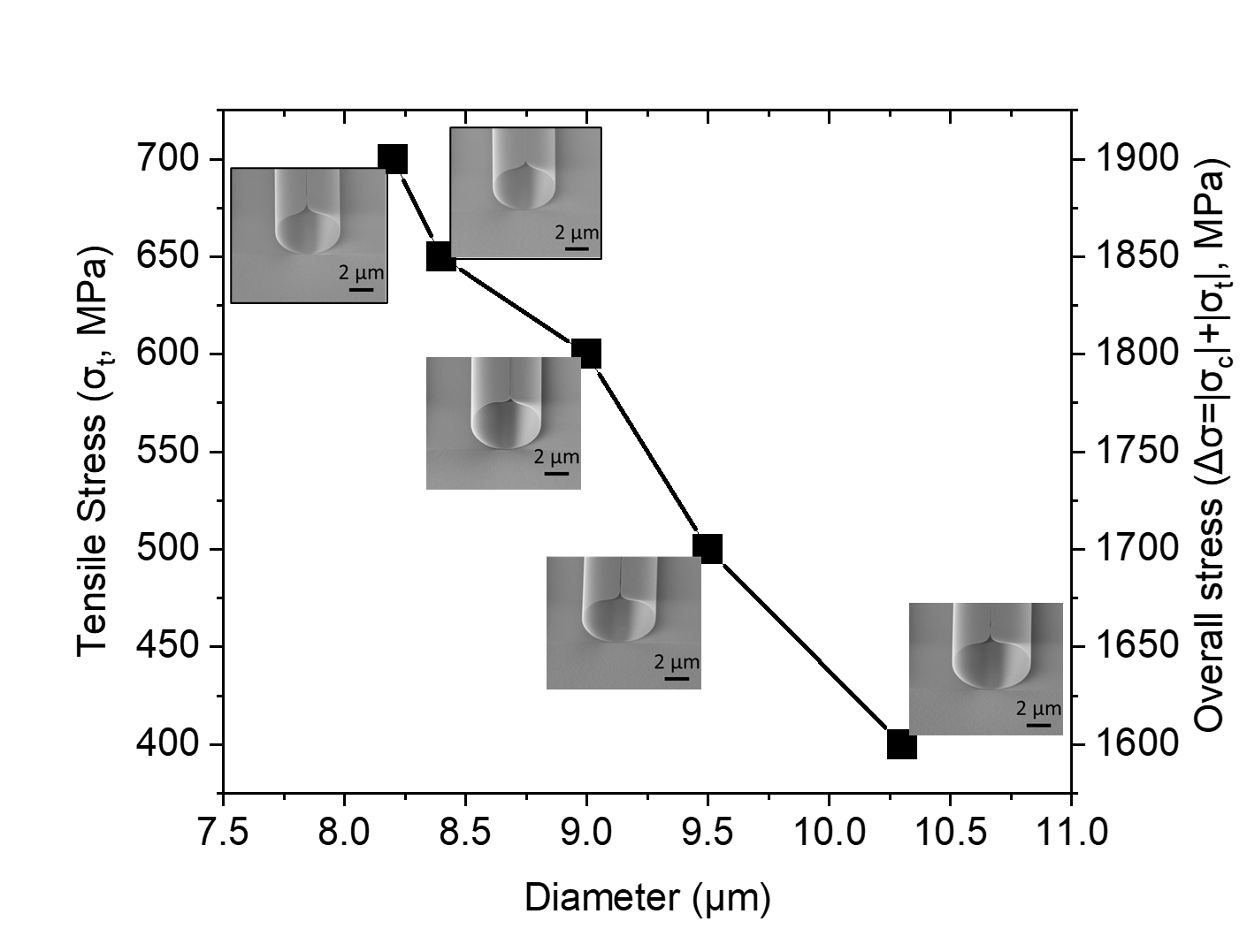


**S2** Plot summarizing results of table S1. Five different bilayer stacks (60 nm thick) with varying tensile stress ($\sigma$_t_) are rolled-up into single turn tubes. The diameter clearly drops as a function of decreasing overall stress (Δσ) in the membranes. The compressive stress ($\sigma$_c_) is maximized and kept constant (-1200 MPa) in all the variations of stress. The overall stress is defined as, $\Delta\sigma$=|$\sigma$_c_|+|$\sigma$_t_|. Attached in the inset of each data point is the zoomed-in SEM image of the single turn microtube for reference.

**Fig. S3: Microfluidic encapsulation for on-chip integrated device.**


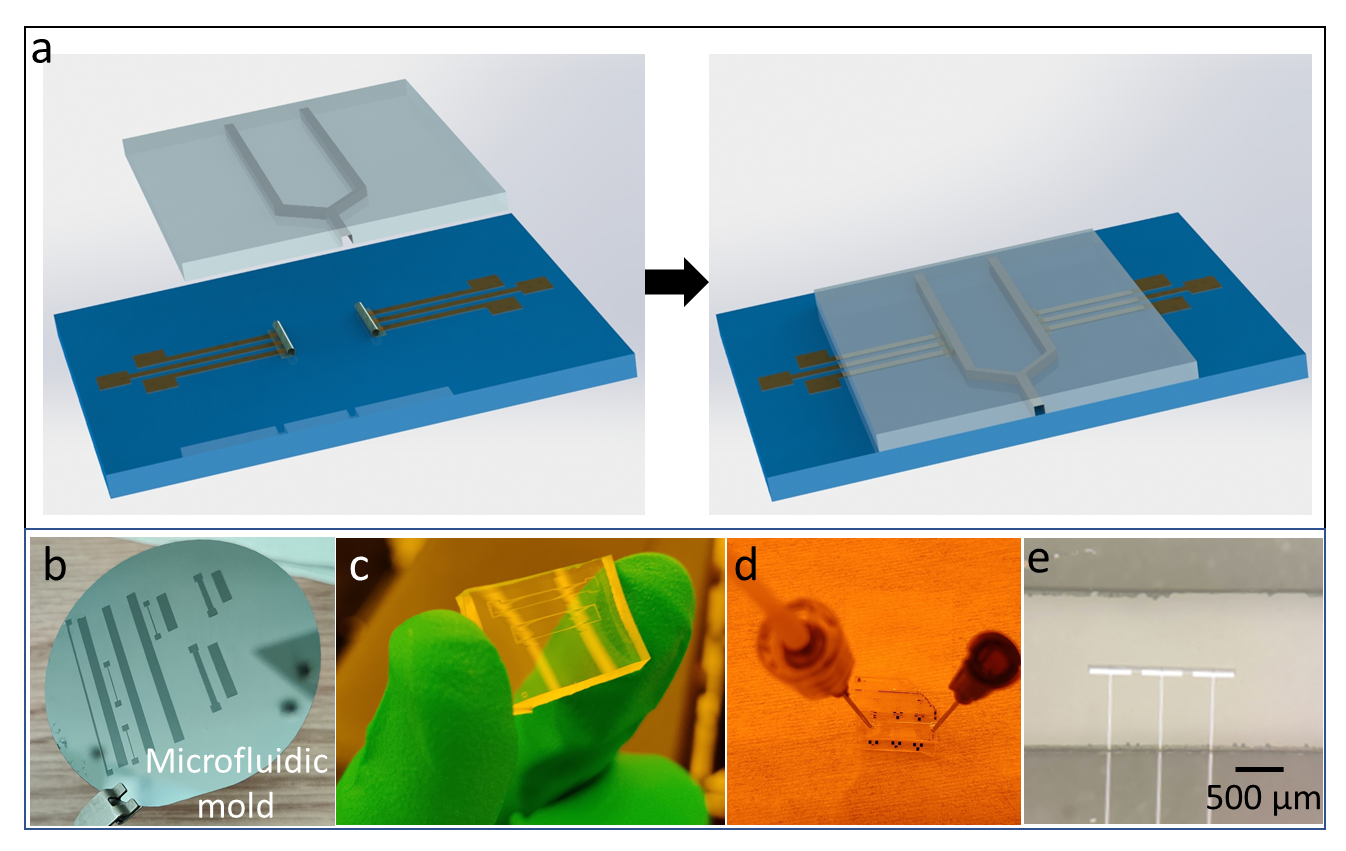


**a**, 3D schematic construction of the microfluidic device. The tubes can be strategically positioned on the substrate to enable linear array integration with traditional microfluidics. The PDMS layer containing channels is aligned and bonded to the substrate containing 3D microtubes. **b**, Optical image of the Si wafer showing fabricated SU-8 mold pattern used for creating channels in PDMS. **c**, Flexible PDMS film with microchannels, **d**, aligned and bonded to the on-chip device. **e**, Optical image of the encapsulated integrated device to be used for in-flow measurements.

**Table S1: Viscosity, volume fraction and the calculated VBN of all liquid components of the microsphere dispersion.**

| Liquid component | Volume fraction | Viscosity, η (mPa.s) | VBN_x_ |
| --- | --- | --- | --- |
| DI Water | 8/15 | 0.89 | -0.0171 |
| Propylene Glycol | 3/15 | 39.436 | 0.3472 |
| Polystyrene sphere solution | 1/15 | 0.89* | -0.0171 |
| IPA | 3/15 | 1.96 | 0.0887 |

***Polystyrene sphere solution is 99% DI water based colloidal solution.**

**Fig. S4: Particle position inside the microtube.**


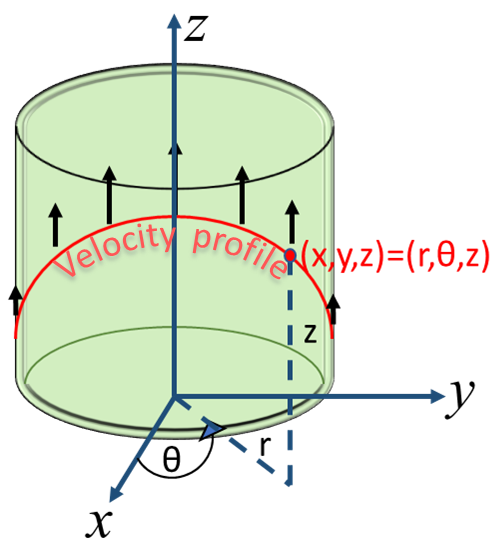


**S3** Cylindrical coordinates or cartesian coordinates can be used to express the instantaneous position of the particle inside the microtube. The Particles traveling along centerline will thus have only z-component. In the x and y direction (radial direction), the electrokinetic particle velocity will vary with the particle–wall separation distance.

**Fig. S5: Electrophoretic mobility of 1µm PSB as a function of applied voltage and generated E-field inside the microtube.**


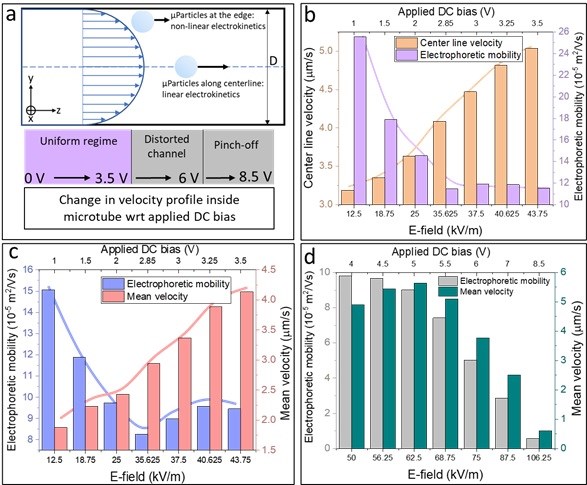


**a**, Schematic illustration of particle dynamics inside the microtube. For particles on the edge, the electrophoretic mobility of the particle will be affected by wall-induced forces. For particles travelling along the centerline (0,0,z) of the microtube, the electrophoretic mobility will be mostly affected by the impressed electrostatic force. **b**, Plot showing flattening of the electrophoretic mobility (based on particles travelling along centerline) curve as the velocity profile becomes more flattened in the regime of 2.85 V to 3.5 V. Also, the centerline particle velocity is linearly increasing with applied voltage (or e-field). **c**, Plot showing the electrophoretic mobility curve (based on mean velocity) showing similar downward trend as the e-field is increased, however, the electrophoretic mobility curve in this case is always dependent on e-field even in the regime of 2.85 V to 3.5 V (flattened velocity profile); showing the effects of non-linear electrokinetics on overall electrophoretic mobility. **d**, Plot showing the electrophoretic mobility trend beyond the uniform regime (>3.5V). In the distorted channel and pinch-off regime, the electrophoretic mobility is strong function of mean particle velocity which is greatly affected by the reverse e-field generated due to accumulated charge and is thus no-longer linearly increasing with applied DC bias.

**Fig. S6: Images showing nanosphere position inside the microtube with respect to applied bias.**

**
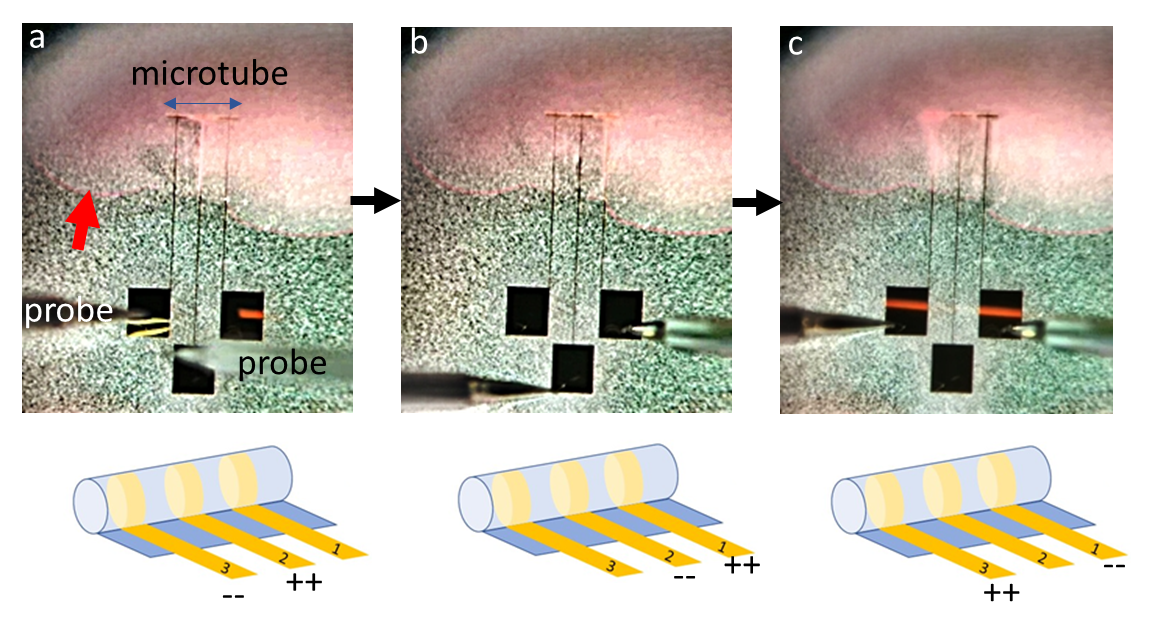
**

**a,** Red arrow marks the droplet boundary. In this case, the middle electrode is biased more positively (as shown in schematic). A bright T-shape formation on the middle electrode in the form of bulk nanosphere accumulation can be seen in the image. Similarly, the nanospheres are then moved to (**b**) right most end (bright pink signal on right) and back to (**c**) left most end (bright pink signal on left) by appropriately biasing the three electrodes (schematic showing: 1= left electrode, 2= middle electrode, 3= right electrode).

**Fig. S7: Fluorescent microscopy images showing areal distribution of nanospheres under applied DC bias.**

**
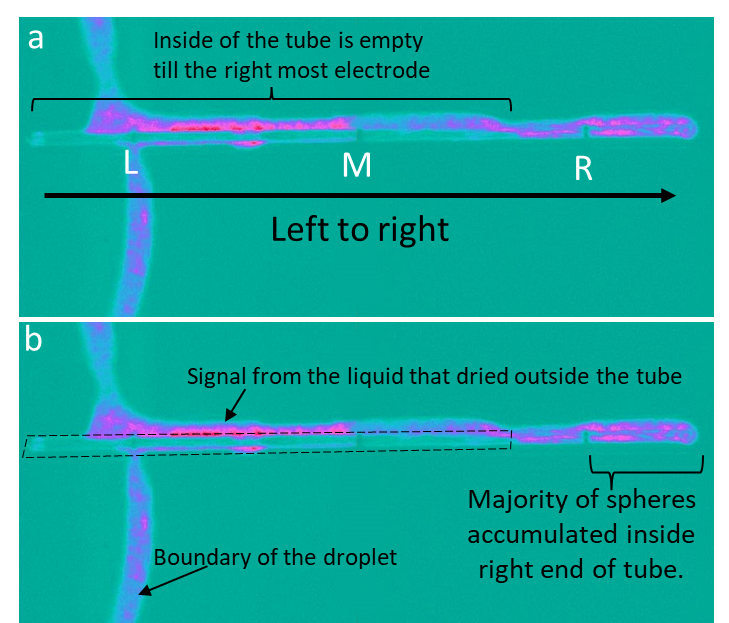
**

**S7** Both figures (**a** and **b)** are same images showing different artifacts related to the imaging. The 2V bias was kept constant until the liquid was dried leaving the nanosphere in their intended locations (in this case to the positively biased right most electrode).

**References**

1. Froeter, P. *et al.* 3D hierarchical architectures based on self-rolled-up silicon nitride membranes. *Nanotechnology* **24**, (2013).

2. Centeno, G., Sánchez-Reyna, G., Ancheyta, J., Muñoz, J. A. D. & Cardona, N. Testing various mixing rules for calculation of viscosity of petroleum blends. *Fuel* **90**, 3561–3570 (2011).

3. Huber, M. L. *et al.* New international formulation for the viscosity of H2 O. *J. Phys. Chem. Ref. Data* **38**, 101–125 (2009).

4. Park, J.-G. *et al.* Interfacial and Electrokinetic Characterization of IPA Solutions Related to Semiconductor Wafer Drying and Cleaning. *J. Electrochem. Soc.* **153**, G811 (2006).

5. Khattab, I. S., Bandarkar, F., Khoubnasabjafari, M. & Jouyban, A. Density, viscosity, surface tension, and molar volume of propylene glycol + water mixtures from 293 to 323 K and correlations by the Jouyban–Acree model. *Arab. J. Chem.* **10**, S71–S75 (2017).

6. Kohlheyer, D., Eijkel, J. C. T., Schlautmann, S., Van Den Berg, A. & Schasfoort, R. B. M. Bubble-free operation of a microfluidic free-flow electrophoresis chip with integrated pt electrodes. *Anal. Chem.* **80**, 4111–4118 (2008).

7. Wereley, S. Infrared Micro-Particle Image Velocimetry Measurement in Silicon-Based Microdevices. (2005).

8. Qian, S. & Ai, Y. Electrokinetic Particle Transport in Micro-/Nanofluidics. *Electrokinet. Part. Transp. Micro-/Nanofluidics* **153**, (2012).

9. Fukasawa, T., Ono, K., Ishigami, T. & Fukui, K. Electrophoretic classification based on differences in electrophoretic mobility caused by change in the applied electric field. *Powder Technol.* **362**, 586–590 (2020).
